# Pan-Cancer Analysis of the Oncogenic and Immunological Role of *RCN3:* A Potential Biomarker for Prognosis and Immunotherapy

**DOI:** 10.3389/fonc.2022.811567

**Published:** 2022-05-16

**Authors:** Jian Ding, Yan Meng, Zelong Han, Xiaobei Luo, Xuxue Guo, Yiwen Li, Side Liu, Kangmin Zhuang

**Affiliations:** ^1^Guangdong Provincial Key Laboratory of Gastroenterology, Department of Gastroenterology, Nanfang Hospital, Southern Medical University, Guangzhou, China; ^2^Pazhou Lab, Guangzhou, China

**Keywords:** pan-cancer, RCN3, immunotherapy, prognosis, potential biomarker

## Abstract

Despite emerging publications have elucidated a functional association between *RCN3* and tumors, no evidence about a pan-cancer analysis of *RCN3* is available. Our study first conducted a comprehensive assessment of its expression profiles, prognosis value, immune infiltration, and relevant cellular pathways *via* bioinformatics techniques based on the public database of TCGA (The Cancer Genome Atlas). *RCN3* is highly expressed in most tumors, and it is associated with poor prognosis. Kaplan-Meier analysis and Cox regression analysis suggested that the high expression of *RCN3* was associated with poor overall survival (OS) in pan-cancer, Cox regression analysis also indicated high *RCN3* expression was correlated with disease-specific survival (DSS) and progression-free interval (PFI) in most tumors. We observed a regulation function of *RCN3* at genetic and epigenetic levels through CNA and DNA methylation using cBioPortal database. Based on Gene Set Enrichment Analysis, we first identified related pathways of *RCN3* and its potential biological functions in pan-cancer, *RCN3* was implicated in oncogenic pathways, and was related to extracellular matrix and immune regulation. We found that *RCN3* positively correlated with the levels of infiltrating cells such as TAMs and CAFs, but negatively correlated with CD8^+^ T-cells by analyzing immune cell infiltration data we downloaded from published work and online databases, further investigation of the correlation between immunosuppressive genes, chemokines, chemokines receptors, and high *RCN3* expression showed a significant positive association in the vast majority of TCGA cancer types. These results indicated its role as an immune regulatory in cancers and suggested that RCN3 is a potential biomarker for immunotherapy. Also, we found that expression of RCN3 was much higher in CRC tissues than in normal tissues with a higher expression level of RCN3 closely correlating to advanced American Joint Committee on Cancer (AJCC) stage, poor differentiation, increased tumor size, and poor prognosis of CRC. Biological function experiments showed that RCN3 regulated CRC cells’ proliferation and metastasis ability. Upregulation of RCN3 in CRC cells increased the expression of immune related factor, including TGFβ1, IL-10, and IL-6. Thus, our pan-cancer analysis offers a deep understanding of potential oncogenic roles of *RCN3* in different cancers.

## Introduction

Cancer is a major public health problem (1). Tumor microenvironment (TME) and tumor-infiltrating immune cells have been reported to be closely associated with cancer initiation, progression, and metastasis. Immunosuppression is one of the notable characteristics of TME (2–5). Although breakthroughs have been made in tumor immunotherapy, which holds promise for the clinical cure of patients with malignant tumors, immunotherapy is still not applicable to all patients (6–9). Therefore, finding novel therapeutic biomarkers and strategies is of seminal importance.

Reticulocalbin 3 (*RCN3*) is a member of the CREC (Cab45/reticulocalbin/ERC45/calumenin) family of multiple EF-hand Ca2+-binding proteins (10, 11). Previous studies suggested that *RCN3* functions as an endoplasmic reticulum (ER) lumen protein in the secretory pathway (12, 13). Many studies have revealed that *RCN3* participates in biological processes such as apoptosis, ER stress, and collagen fibrillogenesis in tissues producing an extracellular matrix (14–16). In addition, limited studies reported that *RCN3* also plays a role in non-small cell lung cancer (NSCLC), melanoma, osteosarcomas, gliomas, and colorectal cancer (17–21). Non-tumor cells like fibroblasts, immune cells in the TME, participate in the tumor biology process through complex interactions with cancer cells (22), previous research has found that fibroblasts with somatic copy number alterations may interact with cancer cells to play synergetic roles in tumor development, and according to the published single-cell multiomics sequencing results, *RCN3* is identified as a fibroblast-specific biomarker of poorer prognosis of colorectal cancer (CRC) (21).These results indicated that RCN3 may act as an essential regulator in tumor progression and immunomodulation. However, there is no evidence focused on the association between *RCN3* and other tumors, and *RCN3* expression in pan-cancer remains unclear.

Our study, for the first time, comprehensively assessed the role of *RCN3* in TCGA pan-cancer. We conducted a pan-cancer analysis to explore *RCN3* expression signature, genetic alteration characteristics, DNA methylation, prognostic value, and immune regulation relevant pathways. We further investigated the correlation between *RCN3* expression with tumor-infiltrating immune cells and immunosuppression-related genes in pan-cancer samples. Our research highlighted the significance of *RCN3* in pan-cancer and its potential oncogenic role in influencing immune infiltration and tumor immunosuppressive microenvironment.

## Materials and Methods

### *RCN3* Gene Expression Analysis

*RCN3* gene expression data in pan-cancer were obtained from the Tumor Immune Estimation Resource, version 2 (TIMER2) database (http://timer.comp-genomics.org/) (23). We utilized the “ggradar” R package to obtain data on *RCN3* expression in 33 TCGA cancer tumors. We downloaded the Genotype-Tissue Expression (GTEx) data from UCSC XENA web server (https://xenabrowser.net/) to obtain data on *RCN3* gene expression in 31 normal tissues. Furthermore, we applied the “ggpubr” R package to obtain box plots of the *RCN3* expression difference between tumor tissues and matched normal tissues of the TCGA project (**Supplemental Table S2**). Box plots of the *RCN3* expression difference in different cancer pathological stages were built based on the “ggplot” R package, which analyzed different clinical stages (stage I, stage II, stage III, and stage IV) of TCGA tumors. Additionally, we employed the UALCAN portal (http://ualcan.path.uab.edu/index.html), a website for analyzing cancer omics data including protein expression analysis (24) to profile the expression of the RCN3 total protein between normal samples and tumor samples of breast cancer, colon cancer, ovarian cancer, clear cell RCC (renal cell carcinoma) and UCEC (uterine corpus endometrial carcinoma). TCGA data for pan-cancer analysis of *RCN3* were all derived from the UCSC XENA web server.

### Genetic Alteration Analysis

The cBioPortal web (https://www.cbioportal.org/) (25, 26), provided us information about genetic alteration characteristics of *RCN3*. We investigated the alteration frequency, mutation type, and CNA (copy number alteration) of *RCN3* in different TCGA tumors. We also used the cBioPortal web to obtain the data on DNA methylation levels of the *RCN3* in various cancers.

### Survival Prognosis Analysis

The relationship between the expression levels of *RCN3* and patients’ overall survival across different types of cancer was evaluated by Kaplan-Meier survival analysis. The noteworthy results were visualized as survival curves built using “survminer” and “survival” R packages. The median cutoff values were used as the expression thresholds for dividing the high and low expression groups. Moreover, we also analyzed patients’ prognosis, including OS (overall survival), DFI (disease-free interval), PFI (progression-free interval), and DSS (disease-specific survival) in 33, 28, 32, and 32 TCGA cancer cases, respectively, *via* univariate Cox proportional-hazards regression analysis. The noteworthy results shown as forest plots were visualized with “survminer” and “survival” R packages. A log-rank P value < 0.05 was considered significant for survival-relevant analysis.

### Gene Set Enrichment Analysis

Gene Set Enrichment Analysis (GSEA) is a commonly used bioinformatics method that conducts genomes enrichment analyses and searches functional terms and pathways (27). In the present study, we employed GSEA to further explore the potential functions of *RCN3* in different cancers. “clusterprofiler” R package was used to perform GO (Gene Ontology) and KEGG (Kyoto Encyclopedia of Genes and Genomes) enrichment analysis. P < 0.05 was considered to be statistically significant. The top 20 categories of each cancer are displayed.

### Immune Infiltration Analysis

TIMER2 web server is a commonly used web resource for analyzing tumor-infiltrating immune cells in various cancers (23). We evaluated the association between *RCN3* expression levels and immune infiltrates across TCGA tumors, including cancer-associated fibroblasts, immune cells of CD8^+^ T-cells, and tumor-associated macrophages. The TIMER, EPIC, XCELL, MCPCOUNTER, TIDE, CIBERSORT, CIBERSORT-ABS, and QUANTISEQ algorithms were applied for the estimations. The data were displayed as a heat map and P < 0.05 was considered to be statistically significant. We then downloaded the TCGA expression data samples from previous studies (28). The CIBERSORT tool (29) was used to assess the correlation between the *RCN3* expression levels and macrophages levels. The scatterplot data presented the P-values and correlation coefficient. Furthermore, a heat map, which contains correlation coefficient and P-value *via* Pearson’s correlation test, was applied to construct correlation analysis between *RCN3* with tumor immunosuppression-related genes, chemokines, and chemokines receptors.

### Tissue Microarray and Immunohistochemistry

The TMA containing a total of 89 colon cancer patients, together with the data of pathological staging in accordance with TNM classification of the American Joint Committee on Cancer (AJCC, 2010) and overall survival time for all cases (**Supplemental Table S1**) was obtained from the National Engineering Center for Biochip at Shanghai. The IHC staining of *RCN3* with scoring was done as below. The section of TMA was baked at 65°C for 30 min. Then the section was deparaffinized with xylenes and rehydrated. After treatment with 3% hydrogen peroxide in methanol to quench the endogenous peroxidase activity, the section was submerged into citrate buffer and high-pressure boiled for antigenic retrieval, followed by incubation with 1% bovine serum albumin to block the nonspecific binding. Rabbit anti-*RCN3* (1:100; Abcam, Cambridge, MA) was incubated with the section overnight at 4°C. After washing, the TMA section were treated with anti-rabbit secondary antibody (Zhongshan Biotech, Beijing, China). The tissue section was incubated with 3,3-diaminobenzidin (DAB) and counterstained with hematoxylin, dehydrated, and mounted. The section was reviewed and scored independently by two observers, based on both the proportion of positively stained tumor cells and the intensity of staining. The proportion of positive tumor cells was scored as follows: 0 (no positive tumor cells), 1 (<10% positive tumor cells), 2 (10–50% positive tumor cells), and 3 (>50% positive tumor cells). The intensity of staining was graded according to the following criteria: 0 (no staining); 1 (weak staining = light yellow), 2 (moderate staining = yellow brown), and 3 (strong staining = brown). The staining index (SI) was calculated as staining intensity score x proportion of positive tumor cells. Using this method of assessment, the expression of *RCN3* was scored as 0, 1, 2, 3, 4, 6, and 9. Cutoff values for *RCN3* were chosen based on a measure of heterogeneity by the log-rank test statistical analysis regarding overall survival. An optimal cutoff value was identified: the score of ≥4 was used to define tumors as high RCN3 expression and ≤3 as low expression of RCN3.

### *In Vitro* Cellular Experiment

Human colorectal cancer cell line HCT116 was cultured in RPMI-1640 medium supplemented with 10% fetal bovine serum and 1% penicillin streptomycin in a humidified atmosphere at 37°C in 5% CO_2_. *In vitro* cellular experiment was shown as follows:

Cell proliferation assay: The stable cell lines were seeded on 96-well plates at an initial density of (1-2×103/well). At each time point, cells were detected by the Cell Counting Kit-8 kit following the kit assay protocol.

Colony formation assays: Cells were seeded on a 6-well plate (200 cells/well) and incubated at 37°C in a humidified 5% CO_2_ atmosphere incubator. After 2 weeks, cells were fixed and stained with hematoxylin. Colonies containing more than 50 cells were counted. Three independent experiments were performed for each cell line.

Cell invasion assays: For invasion assays, the membrane was covered with 40 μl of the BD Matrigel (diluted 1:8 with serum-free medium) in advance. The stable cells (1–1.5 × 105) were seeded into the transwell chambers with serum-free medium while the lower chamber was covered with 500 μl medium supplemented with 20% fetal calf serum. After 48 h incubation at 37°C, cells were harvested and fixed in 4% paraformaldehyde for 20 min and then stained with 0.5% crystal violet diluted in methanol for 25 min. The membrane was removed and mounted onto glass slides and counted (5 × 200 random fields per membrane). Three independent experiments were performed, and the data are presented as mean ± S.D.

## Results

### *RCN3* Expression Levels in Human Cancers

**Figure 1:**
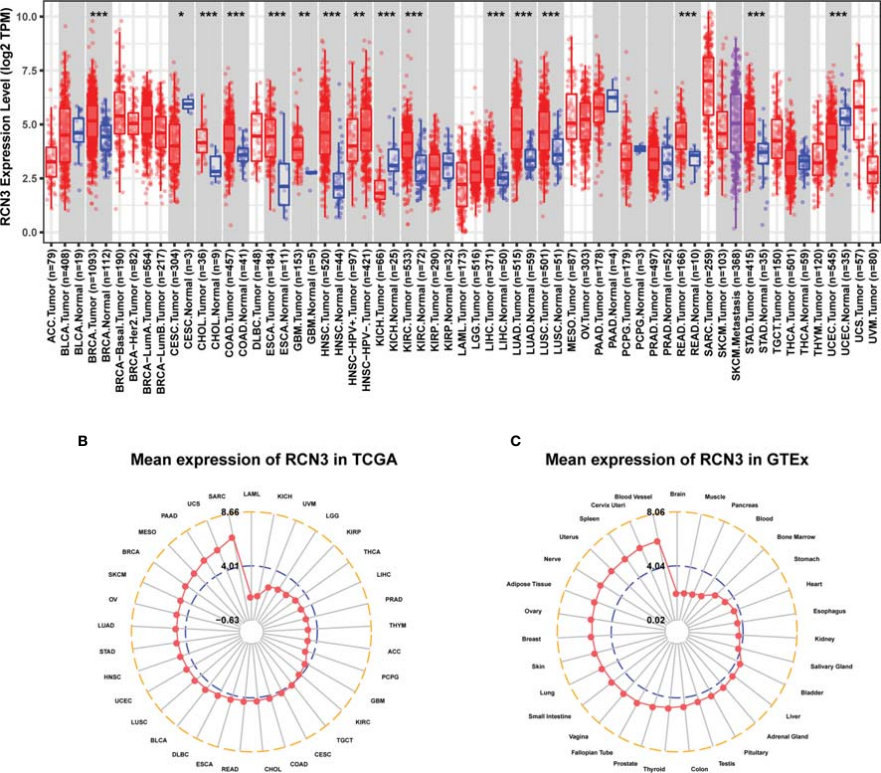
RCN3 expression levels in different human cancers. **(A)** The expression level of RCN3 in different tumor tissues and normal tissues was analyzed through TIMER2 database (*P < .05, **P < .01, ***P < .001). The radar charts (ggradar package was used for analysis) showing the expression of RCN3 in different cancers from TCGA **(B)** and in normal tissues of the GTEx **(C)**. Mean expression was the basis of ranking.

We first analyzed the expression characteristics of *RCN3* in tumor and normal tissues from TCGA *via* TIMER2 database. As shown in **Figure 1A**, up-expression of *RCN3* was observed in most cancer types, such as LUSC (lung squamous cell carcinoma), LUAD (lung adenocarcinoma), LIHC (liver hepatocellular carcinoma), KIRC (kidney renal clear cell carcinoma), HNSC (head and neck squamous cell carcinoma), ESCA (esophageal carcinoma), COAD (colon adenocarcinoma), CHOL (cholangiocarcinoma), BRAC (breast invasive carcinoma), and GBM (glioblastoma multiforme). In contrast, the expression of *RCN3* was below that in normal control tissues in KICH (kidney chromophobe) and CESC (cervical squamous cell carcinoma and endocervical adenocarcinoma). Furthermore, we evaluated the expression levels of *RCN3* in different tumor tissues and matched normal tissues using the ggpubr package. *RCN3* was found to be overexpressed in BRCA, CHOL, COAD, ESCA, HNSC, KIRC, LUAD, LUSC, and STAD (stomach adenocarcinoma) but was underexpressed in KICH (**Supplementary Figures 1A–J**). Meanwhile, the expression of *RCN3* was analyzed using the data of multiple types of cancers downloaded from TCGA (**Figure 1B**). Next, we analyzed *RCN3* expression in the 31 types of normal tissues through the GTEx dataset, which revealed *RCN3* was generally expressed in various tissues (**Figure 1C**). Collectively, these results indicated that *RCN3* has an elevated expression in most cancers.

**Figure 2:**
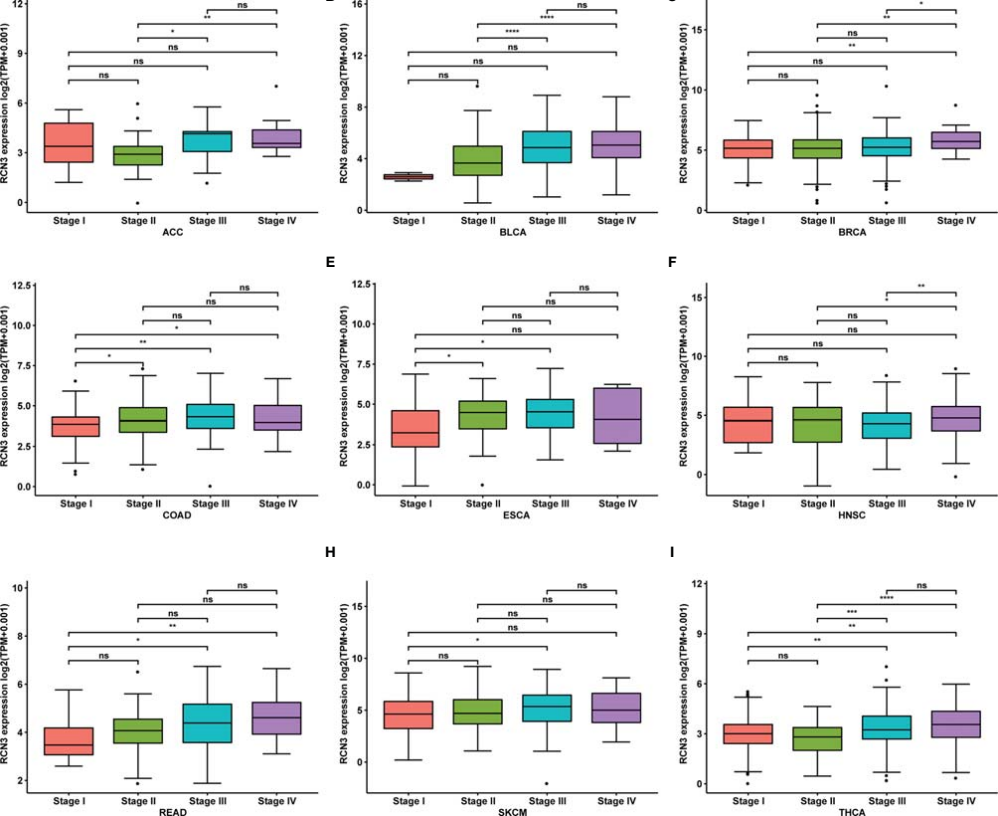
Correlation between RCN3 expression and pathological stages of cancers in TCGA. **(A–I)** Box plot showing the relationship of different tumor stages with RCN3 expression in different cancers from TCGA. *p < 0.05; **p < 0.01; ***p < 0.001; ****p < 0.0001; ns, no significance.

We then explored mRNA expression patterns of *RCN3* in different clinical stages. As shown in **Figures 2A–I**, *RCN3* expression varied significantly in different pathological stages of cancers, including ACC (adrenocortical carcinoma), BLCA (bladder urothelial carcinoma), BRCA, COAD, ESCA, HNSC, READ (rectum adenocarcinoma), SKCM (skin cutaneous melanoma), and THCA (thyroid carcinoma).

These findings suggest that *RCN3* expression levels were higher in higher clinical stages.

### CNA and DNA Methylation Alternations of *RCN3* in Different Cancers

**Figure 3:**
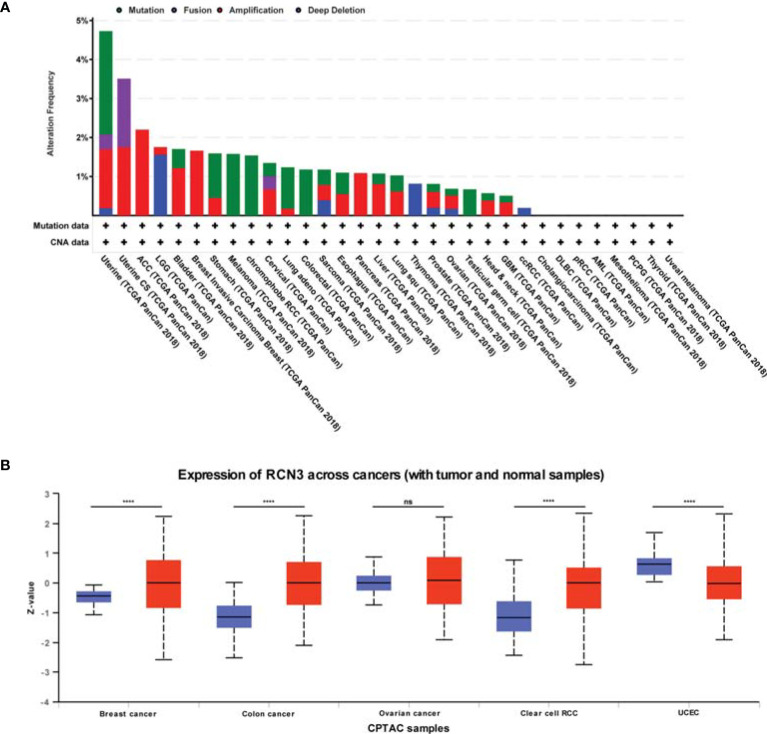
CNA and total protein expression levels of RCN3 in human cancers. **(A)** CNA and mutation frequency data of RCN3 for the TCGA tumors were accessed using the cBioPortal tool. **(B)** The expression of the RCN3 total protein between normal samples and tumor samples of breast cancer, colon cancer, ovarian cancer, clear cell RCC, and UCEC were determined by UALCAN. ****p < 0.0001; ns, no significance.

We observed the genetic alteration status of *RCN3* using cBioPortal. The highest alteration frequency of *RCN3* (>4%) appears for patients with uterine cancers, in which “mutation” is the primary genetic alteration type (**Figure 3A**). It is worth nothing that all ACC, breast invasive cases, and pancreas tumor cases with gene alteration (>2%, >1%, >1% frequency, respectively) had copy number amplification of *RCN3* (**Figure 3A**). We applied the cBioportal data bank to analyze the relationship between *RCN3* expression and relative linear copy-number values. As shown in **Supplementary Figure 3**, a significant positive correlation was found between *RCN3* expression and CNA in READ, SARC (sarcoma), UCEC (uterine corpus endometrial carcinoma), UCS (uterine carcinosarcoma), UVM (uveal melanoma), GBM, LGG (brain lower grade glioma), MESO (mesothelioma), and OV (ovarian serous cystadenocarcinoma). After exploring gene expression variation contributed by CNA, we then investigated whether expression of *RCN3* was regulated by epigenetic mechanisms, such as DNA methylation. We used the cBioportal data bank to explore the correlations between *RCN3* expression and promoter DNA methylation levels, the results revealed a significant negative correlation between *RCN3* expression and DNA methylation in BLCA, BRCA, CESC, CHOL, LIHC, ESCA, HNSC, KIRC, LIHC, LUAD, LUSC, ESO, PAAD (pancreatic adenocarcinoma), PRAD (prostate adenocarcinoma), READ, SARC, SKCM, STAD, UCEC and UCS (**Supplementary Figure 2**). Therefore, both genetic alterations and lower DNA methylation levels are likely the contributed factors for the abnormal elevate of *RCN3* in most cancers.

We also used the UALCAN tool to determine the expression of the RCN3 total protein between different tumors and normal samples. The results showed higher expression of RCN3 total protein in tumor samples of colon cancer, breast cancer, and clear cell RCC than in normal samples but the opposite result was obtained in UCEC (**Figure 3B**).

### Prognostic Significance of *RCN3* Expression in Pan-Cancer

**Figure 4:**
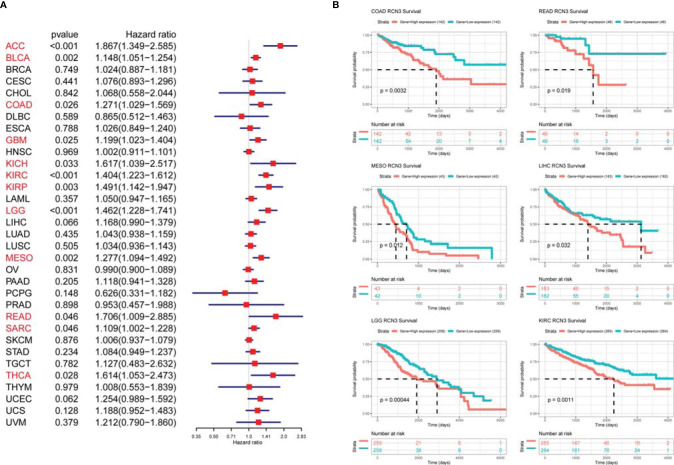
Relationship of RCN3 expression with survival prognosis in pan-cancer. **(A)** Univariate Cox proportional-hazards regression used to quantify the relationship of RCN3 with overall survival. High RCN3 expression correlated with bad prognosis in ACC, BLCA, COAD, GBM, KICH, KIRC, KIRP, LGG, MESO, READ, SARC, and THCA (HR>1, p-value<0.05). **(B)** Kaplan-Meier curves showing higher expression of RCN3 was related to poor prognosis of OS in 6 cancer types. The survival curves with logrank p<0.05 are given.

We applied univariate Cox proportional-hazards regression analysis to quantify the relationship between *RCN3* expression and overall survival in various cancers. High *RCN3* expression was associated with bad prognosis in ACC, BLCA, COAD, GBM, KICH, KIRC, KIRP, LGG, MESO, READ, SARC, and THCA (OS: HR>1, p-value<0.05, **Figure 4A**). Also, the Kaplan-Meier analysis results indicated that higher expression of *RCN3* was related to poor prognosis of OS in COAD, READ, MESO, LIHC, LGG, and KIRC (**Figure 4B**). Furthermore, our results showed that high *RCN3* expression indicated poor DFI in ACC, BRCA, and KIRP (**Supplementary Figure 4A**); poor PFI in ACC, BLCA, BRCA, KICH, KIRC, KIRP, LGG, MESO, and PRAD (**Supplementary Figure 4B**); and poor DSS in ACC, BLCA, BRCA, COAD, GBM, KICH, KIRC, KIRP, LGG, and MESO (**Supplementary Figure 4C**). Also, we found that high expression of RCN3 shared a higher nodal metastasis stage in ACC, COAD, BRCA, and READ (**Supplementary Figures 5A–D**). A GSVA analysis of 32 cancer types showed that expression of RCN3 significantly correlated with the pathway of epithelial mesenchymal transition (EMT) (**Supplementary Figure 5E**). These findings suggest that high *RCN3* expression was generally associated with poor outcomes in pan-cancer analysis.

**Figure 5:**
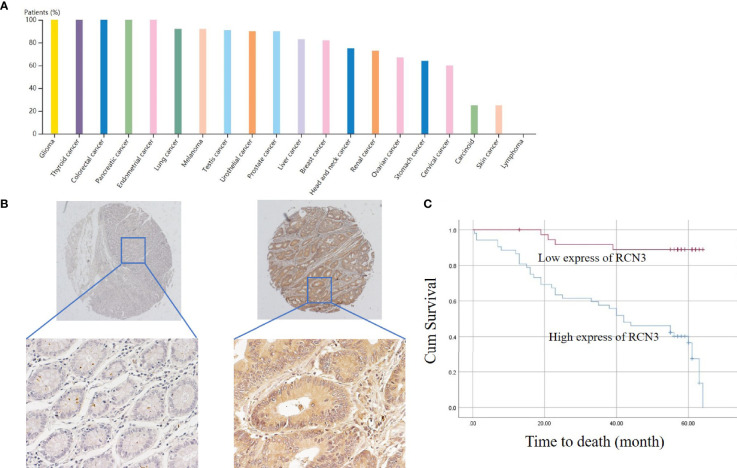
Protein expression of RCN3 in pan-cancer and its prognosis role in CRC. **(A)** Protein expression of RCN3 in pan-cancer was shown by the human protein atlas. **(B)** The protein level of RCN3 was found highly expressed in the TMA containing 89 colon cancer cases. **(C)** Kaplan–Meier overall survival curves for patients with CRC stratified by low (n = 37) and high (n = 52) expression of RCN3 (P = 0.000).

Furthermore, the protein expression level of RCN3 was shown by the *human protein atlas*, in which we found that the protein level of RCN3 was significantly expressed in most types of cancer, with glioma, thyroid cancer, and colorectal cancer ranked in the top 3 (**Figure 5A**). To further identify the prognostic significance of RCN3, a TMA of colorectal cancer was carried out. As a result, RCN3 protein was detected in 84 of 89 (94.4%) cases of CRC samples with 52 (61.9%) cases displaying high expression (**Table 1** and **Figure 5B**). Besides, Pearson chi-square tests revealed that the expression of RCN3 was significantly correlated with AJCC (*P*=0.012) tumor size (*P*=0.002) and nodal metastasis (*P*=0.002) (**Table 1**). Notably, Kaplan-Meier survival analysis revealed that patients with high RCN3 expression had poorer overall survival than patients with low RCN3 expression (39.7 vs. 59.7 months, *P*=0.000) (**Figure 5C**). These findings strongly suggest that RCN3 may serve as a potential oncogenic role in CRC.

**Table 1:** Correlation between clinicopathological features and RCN3 expression levels.

Variable	n	RCN3 expression		χ^2a^	*P*
		Low	High		
**Gender**				0.005	0.946
Male	46	20	26		
Female	43	19	24		
**Age (y)**				0.096	0.757
<59	26	9	17		
≥59	63	24	39		
**Tumor size (cm in diameter)**				9.488	**0.002**
<5	32	19	13		
≥5	57	15	42		
**Nodal metastasis**				9.561	**0.002**
N0	48	31	17		
N1/2	41	13	28		
**AJCC Stage**				6.273	**0.012**
I/II	53	32	21		
III/IV	36	12	24		

^a^Pearson Chi-square was used for assay. P< 0.05, the difference was statistically significant in bold text.

### Enrichment Analysis of *RCN3*-Related Pathways in Human Cancers

**Figure 6:**
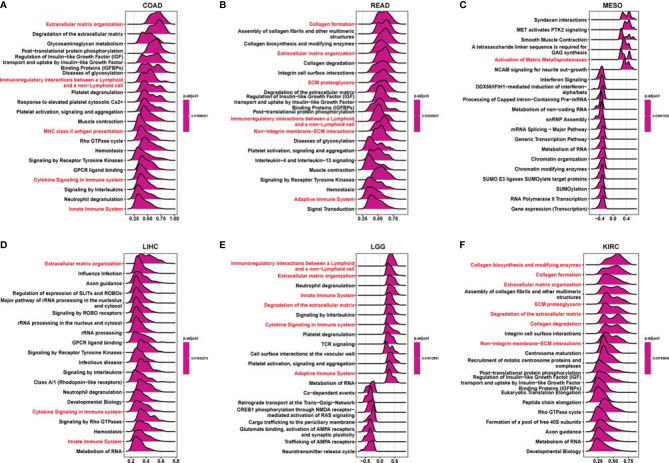
Gene Set Enrichment Analysis (GSEA) of tumor samples with expression of RCN3. **(A–F)** The plot showing the top 20 categories enriched in GO analysis *via* GSEA. The extracellular matrix related pathways and immune-related regulation mechanisms (red) were the main enriched terms in a large fraction of cancers, such as COAD, READ, MESO, LIHC, LGG, and KIRC.

GSEA was performed to further explore the potential biological functions of *RCN3* in different cancers. The top 20 categories of each cancer are displayed. The GO GESA results indicated the enrichment in many categories and *RCN3* had a main effect on terms about the extracellular matrix-related processes such as “extracellular matrix organization”, “collagen formation”, “ECM proteoglycans”, “non-integrin membrane-ECM interaction”, and “degradation of the extracellular matrix” and immune-related regulation mechanisms such as “cytokine signaling in immune system”, “immunoregulatory interactions between a lymphoid and a non-lymphoid cell”, “innate immune system”, “adaptive immune system”, and “MHC class II antigen presentation” in a large fraction of cancers, including COAD, READ, MESO, LIHC, LGG, and KIRC (**Figure 6**). KEGG GSEA analysis demonstrated that *RCN3* was mainly involved in “ECM − receptor interaction”, “cytokine-cytokine receptor interaction”, “focal adhesion”, “PI3K − Akt signaling pathway”, “chemokine signaling pathway”, “pathways in cancer”, and “Th1 and Th2 cell differentiation” in COAD, READ, MESO, LIHC, LGG, and KIRC (**Supplementary Figure 6**).

### Correlation Analysis of *RCN3* Expression and Immune Infiltration Levels Across Cancers

**Figure 7:**
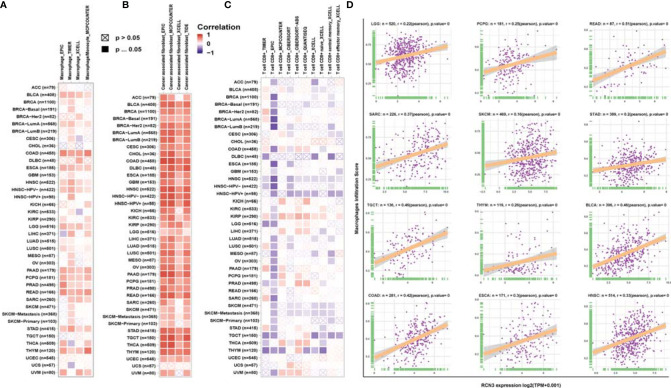
Relationship between RCN3 expression and infiltration levels of immune cells. Based on the TIMER2 database, we analyzed the potential correlation between the expression level of the RCN3 and the infiltration levels of tumor-associated macrophage **(A)**, cancer-associated fibroblasts **(B)**, and CD8+ T cells **(C)**. **(D)** Correlation analysis of RCN3 expression with macrophages infiltration score in cancers from TCGA by CIBERSORT.

Tumor-infiltrating immune cells in the tumor microenvironment had a close connection with the tumor biology process and cancer patients’ survival (4). Evidence suggests that cancer-associated fibroblasts in the TME affected the function of various tumor-infiltrating immune cells (30–32). Therefore, TIMER, EPIC, XCELL, MCPCOUNTER, TIDE, CIBERSORT, CIBERSORT-ABS, and QUANTISEQ algorithms were used to identify the potential correlation between *RCN3* expression and the infiltration level of immune cells in human pan-cancer. We found a statistical positive correlation between the macrophages and *RCN3* expression in tumors of BLCA, BRCA-LumA, COAD, ESCA, HNSC, HNSC-HPV-, LGG, PAAD, PCPG, PRAD, READ, STAD, and THYM (**Figure 7A**) *via* all or most algorithms we used. We also observed a significant positive correlation of *RCN3* expression and the infiltration level of cancer-associated fibroblasts in diverse cancer types of TCGA (**Figure 7B**) *via* all or most algorithms we used. Moreover, considering macrophages’ function in suppressing T cells, we investigated the relationship between *RCN3* and CD8^+^ T cells. Our results showed that *RCN3* expression level was negatively correlated with the level of immune infiltration of CD8^+^ T-cells in the tumors of HNSC, HNSC-HPV^+^, SKCM, and SKCM-metastasis. (**Figure 7C**). Moreover, the cell-specific expression of RCN3 was also derived using the UALCAN database and found that notheness in solid or nonsolid tumor cells, RCN3 showed a high expression in immune cells and fibroblast cells (**Supplementary Figure 7**). These results indicated that *RCN3* may play an important role in regulating tumor immunosuppressive microenvironment in pan-cancer, with a positive correlation with macrophages and cancer-association fibroblasts but a negative correlation with CD8^+^ T-cells.

To further verify the relationship between *RCN3* expression and infiltration levels of macrophages, correlation analyses were conducted using immune cell infiltration data we downloaded from published work (28), which evaluated immune cells using the CIBERSORT tool. The scatterplot data presented in **Figure 7D** illustrates that the *RCN3* expression levels were positively correlated with the macrophages infiltration score in a variety of cancers like LGG, PCPG, READ, SARC, SKCM, STAD, TGCT, THYM, and BLCA, which is consistent with the results from TIMER.

### Association of *RCN3* Expression with Immuno-Related Genes

**Figure 8:**
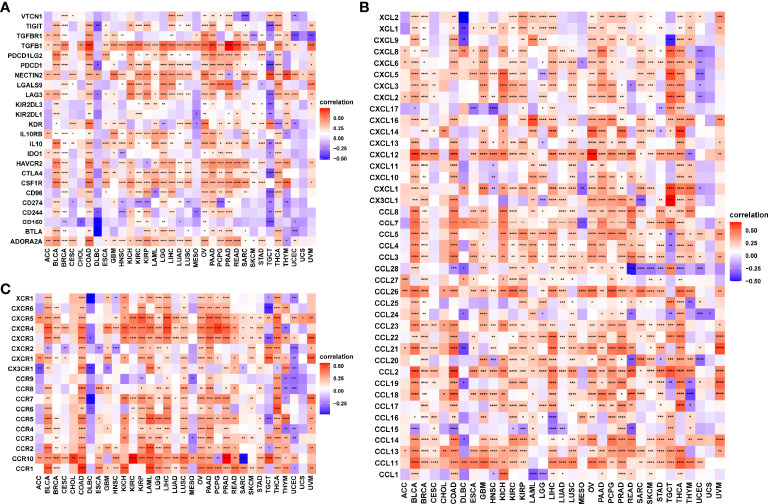
Correlation analysis between RCN3 expression and immuno-related genes. Correlation analysis between RCN3 expression and immunosuppression-related genes **(A)**, chemokines **(B)**, and chemokines receptors **(C)**. *p < 0.05; **p < 0.01; ***p < 0.001; ****p < 0.0001.

Our pan-cancer analysis revealed the significant positive correlations of *RCN3* expression with macrophages in most cancers from TCGA. Herein, we applied correlation analysis between *RCN3* expression and tumor immunosuppression-related genes for further validation. The heat-map (**Figure 8A**) according to the correlation between *RCN3* and multiple immunoinhibitory molecules including TIGIT, TGFB1, PDCD1LG2, PDCD1, NECTIN2, LGALS9, LAG3, IL10RB, IL10, HAVCR2, CTLA4, CSF1R, CD96, BTLA, and ADORA2A in pan-cancer suggested that most correlations were positive. It is essential to note that TGFB1, which is an effector of immune suppression, showed strong positive correlations with *RCN3* expression levels in the vast majority of TCGA cancer types. Moreover, significant correlations were found between almost all tumor immunosuppression-related genes we assessed and *RCN3* in COAD. We also focused on the relationship between *RCN3* expression and chemokines (**Figure 8B**) and chemokines receptors (**Figure 8C**). High *RCN3* expression was positively associated with multiple chemokines, especially CXCR5, CXCR4, CXCR3, CCR10, CCR2, and CCR1. *RCN3* expression had a significant positive association with various chemokine receptors in different cancers as well. Our results suggested the *RCN3* gene plays a vital role in tumor immunity and confirmed that high *RCN3* expression level was positively associated with tumor immunosuppression in multiple cancer types. High *RCN3* expression indicated tumor immune inhibition status may not be conducive to immunotherapy.

### *RCN3* Promoted the Proliferation and Invasion Ability and Increased Expression of Tumor Immune Related Gene of CRC Cells *In Vitro*

**Figure 9:**
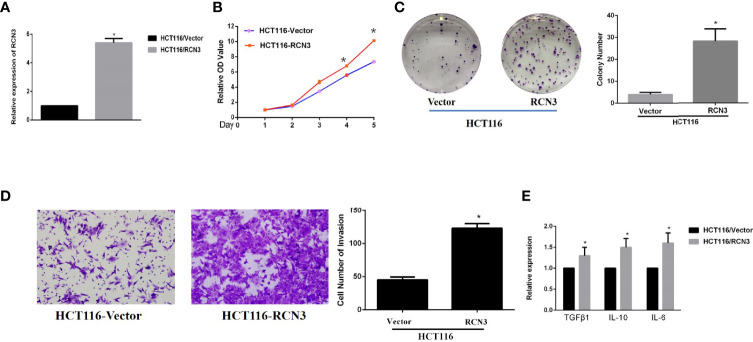
Fold change of expression level of RCN3’s RNA expression after transfection in HCT-116 was shown **(A)**. Over-expression of RCN3 significantly promotes the proliferation and invasion ability in CRC cells **(B-D)** and promotes the expression of immune related factor, including TGFβ1, IL-10, and IL-6 **(E)**. *p < 0.05

Finally, we performed the *in vitro* experiment in CRC cells to further identify the oncogenic role of RCN3. Interestingly, overexpression of *RCN3* exhibited a remarkable promotion of cell proliferation ability and cell colony formation (**Figures 9A, B**). Also, overexpression of RCN3 in HCT116 cells promotes the invasion ability compared with the control group (**Figure 9C**).

Moreover, qRT-PCR experiments of the indicated cell lines showed that overexpression of RCN3 showed a significantly increased level of TGFβ1, IL-10, and IL-6, indicating that RCN3 might promote oncogenic progress of CRC with immunological regulation, which also matched our previous bioinfomatic analysis of its pan-cancer role (**Figure 9D**).

## Discussion

Reticulocalbin 3 (*RCN3*) is known as an endoplasmic reticulum (ER) lumen protein function in the secretory pathway (12, 13). Many studies have revealed that *RCN3* participates in a series of biological processes such as apoptosis, ER stress, and collagen fibrillogenesis in tissues producing extracellular matrix (14–16). Despite emerging publications have elucidated a functional association between *RCN3* and diversity clinical diseases, such as tumors (17–21), there is no evidence about a pan-cancer analysis of *RCN3* based on overall cancers. Whether *RCN3* can affect tumor pathogenesis through certain common molecular mechanisms is not yet fully established. Thus, we comprehensively assessed molecular characteristics of *RCN3* expression, genetic and epigenetic signature, prognostic value, and its potential oncogenic role in regulating immune infiltration and tumor immunosuppressive microenvironment in TCGA pan-cancer.

We first analyzed *RCN3* expression characteristics in tumor and normal tissues from TCGA and GTEx database, *RCN3* was highly expressed in most tumors, such as LUSC, LUAD, LIHC, KIRC, HNSC, ESCA, COAD, CHOL, BRAC, and GBM. In contrast, low *RCN3* expression was observed only in KICH and CESC. We also detected a correlation between high expression levels of *RCN3* and higher pathological stages in ACC, BLCA, BRCA, COAD, ESCA, HNSC, READ, SKCM, and THCA. It has been reported that *RCN3* is a biomarker of poorer prognosis of colorectal cancer (21). In this study, our analysis of univariate Cox proportional-hazards regression suggested the link between *RCN3* expression and overall survival prognosis in 12 cancer types. Additionally, the Kaplan-Meier analysis also represented a significant correlation between high *RCN3* expression and poor clinical prognosis of OS in COAD, READ, MESO, LIHC, LGG, and KIRC. Furthermore, our results also suggested that high *RCN3* expression indicated poor DFI, PFI, and DSS in different tumors. Consequently, *RCN3* was highly expressed in most cancers and high *RCN3* expression was generally associated with poor outcomes in most cancers. To further identify its prognostic roles, we derived a TAM experiment of CRC tissue and found that expression levels of RCN3 significantly correlated with the aggressive characteristics of the cancer (AJCC stage, tumor size, and nodal metastasis) as well as poor survival of patients. These findings are consistent with the pan-cancer analysis and suggested that RCN3 might be a universal tumor biomarker to identify patients with poor clinical outcomes.

Tumor immunotherapy is a promising approach for the treatment of cancer. Immune checkpoint inhibitors (ICIs), such as anti-CTLA4, anti-PD1, and anti-PDL1, genetically engineered T-cell therapy like CAR T-cell therapy, have improved survival in tumor patients. However, the anti-tumor efficacy remains limited in clinical applications (33–35). One of the major challenges rendering tumors resistant is the aberrant tumor microenvironment (TME). The main components of infiltrating stromal cells in the TME are tumor-associated macrophages (TAMs) and cancer-associated fibroblasts (CAFs), and they are reported to play a vital role in tumor progression and chemo-resistance (36). A previous article reported that fibroblasts with somatic copy number alterations in the TME play synergetic roles in tumor development through complex interactions with cancer cells, and *RCN3* is identified as a fibroblast-specific biomarker of poorer prognosis of colorectal cancer (CRC) (21). In contrast, the tumor-infiltrating lymphocytes (TLSs), CD8^+^ T lymphocytes, play a pivotal role in survival prognosis, tumor regression, and antitumor immunity, through weakening CD8^+^ T cell activation, immunosuppressive factors in TME maintain tumor immune inhibition status (37–39). Thus, TAMs and CAFs are potential target cells for cancer treatment. In our study, using the GSEA tool, we integrated the information that *RCN3* had a main effect on the extracellular matrix-related pathways and immune-related regulation mechanisms. Moreover, we identified that *RCN3* expression was positively correlated with TAMs and CAFs but negatively correlated with CD8^+^ T-cells in pan-cancer *via* TIMER2 and analyzed data from a published work. Our results indicated that *RCN3* may play a significant role in regulating tumor immunosuppressive microenvironment in pan-cancer. Furthermore, we investigated the correlations between *RCN3* expression and tumor immunosuppression-related genes, chemokines, and chemokines receptors. Many immunoinhibitory molecules such as TIGIT, TGFB1, PDCD1LG2, PDCD1, NECTIN2, LGALS9, LAG3, IL10RB, IL10, HAVCR2, CTLA4, CSF1R, CD96, BTLA, and ADORA2A were positively correlated with *RCN3* in most cancer types. Chemokines and chemokine receptors such as CCL2, CCL11, CXCR5, CXCR4, CXCR3, CCR10, CCR2, and CCR1 were also positively correlated with *RCN3* expression in most cancer types. The interaction between tumors and tumor immune microenvironment is complicated. Evidence shows that TAMs secret multiple immunosuppressive mediators, such as transforming growth factor (TGF)-B1 and interleukin-10 (IL-10), CCL2-CCR2 pathways, play a crucial role in hindering immunotherapy efficiency tumor and promoting tumor progression (40–43). Our results suggested the *RCN3* gene plays a vital role in tumor immunity and high *RCN3* expression may indicate tumor immune inhibition status.

Genetic and epigenetic alterations are closely linked to tumorigenesis and tumor immunogenicity, for example, dysregulated DNA methylation can lead to aberrant gene expression and promote cancer onset, which meanwhile may vary response to immune regulation in cancer development (44). Using the cBioPortal tool, we observed the genetic alteration status of *RCN3*, the results revealed a significant positive correlation between *RCN3* expression and CNA. Additionally, there was a significant negative correlation between *RCN3* expression and DNA methylation in pan-cancer. To further explore the role of *RCN3* in tumorigenesis and immune regulation, we applied GESA KEGG analysis. The results indicated a correlation between *RCN3* and oncogenic pathways, such as PI3K − Akt, pathways in cancer, and Th1 and Th2 cell differentiation. The precise molecular mechanisms that *RCN3* plays an oncogenic role deserved to be clarified. Our research also has certain limitations. We have limits in our experiments still and the mechanism that can help us better understand the effects of RCN3 both in pan-cancer and colorectal cancer, in which we are interested, remains to be explored. More sufficient *in vivo* and *in vitro* experimental evidence should be provided to verify the oncogenic function of *RCN3* and clinical trials should be performed to determine the role of *RCN3* as an immunotherapeutic biomarker.

In summary, our first pan-cancer analyses explored *RCN3* expression characteristics, prognostic significance, immune cell infiltration, and associated pathways using bioinformatics methods. We conducted a comprehensive analysis of *RCN3*, revealing its potential pro-tumor effect as well as its function of indicating patient prognosis. Importantly, high *RCN3* expression often suggests tumor immune inhibition status, which may not be conducive to immunotherapy. Our research validated the importance of *RCN3* in cancer and a comprehensive understanding of its role as a therapeutic biomarker is worth further exploration.

## Data Availability Statement

The original contributions presented in the study are included in the article/**Supplementary Material**. Further inquiries can be directed to the corresponding authors.

## Author Contributions

KZ and SL designed the study. JD, YM, XL, and ZH recorded the data. XG and YL analyzed the data. JD and YM drafted the manuscript. All authors have read and approved the submitted version of the paper.

## Funding

The project was supported by the National Natural Science Foundation of China (81802965), the Presidential Foundation of Nanfang Hospital, Southern Medical University (Grant No. 2020B020), Guangdong gastrointestinal disease research center (No. 2017B020209003), the National Natural Science Funds of China (12026605).

## Conflict of Interest

The authors declare that the research was conducted in the absence of any commercial or financial relationships that could be construed as a potential conflict of interest.

## Publisher’s Note

All claims expressed in this article are solely those of the authors and do not necessarily represent those of their affiliated organizations, or those of the publisher, the editors and the reviewers. Any product that may be evaluated in this article, or claim that may be made by its manufacturer, is not guaranteed or endorsed by the publisher.

## Supplementary Material

Supplementary Figure 1 The expression level of RCN3 in cancers. **(A–J)** Based on the TCGA data, the expression levels of RCN3 in different tumor tissues and matched normal tissues were analyzed using the ggpubr package.

Supplementary Figure 2 DNA methylation of RCN3 in human cancers. Using the cBioportal tool, we explored the correlations between RCN3 expression and DNA methylation. R>0.3, P<0.05 is considered statistically significant, and only statistical results are shown here.

Supplementary Figure 3 CNA of RCN3 in human cancers. Using the cBioportal data bank, we analyzed the relationship between RCN3 expression and relative linear copy-number values. R>0.2, P<0.05 is considered statistically significant, and only statistical results are shown here.

Supplementary Figure 4 The prognostic significance of RCN3 in pan-cancer. **(A)** Univariate Cox proportional-hazards regression used to quantify the relationship of RCN3 with DFI **(A)**, PFI **(B)**, and DSS **(C)**. DFI, disease-free interval; PFI, progression-free interval; DSS, disease-specific survival.

Supplementary Figure 5 Correlation ship of RCN3 and tumor metastasis. High expression of RCN3 share a higher nodal metastasis stage in ACC, COAD, BRCA, and READ **(A–D)**, and a GSVA analysis of 32 cancer types showed that expression of RCN3 significantly correlated with the pathway of epithelial mesenchymal transition (EMT) **(E)**.

Supplementary Figure 6 Gene Set Enrichment Analysis (GSEA) of RCN3 in pan-cancer. **(A–F)** The plot shows the top 20 pathways enriched in KEGG analysis *via* GSEA. Significant KEGG pathways influenced by RCN3 expression in COAD, READ, MESO, LIHC, LGG, and KIRC were analyzed by GSEA.

Supplementary Figure 7 Cell-specific expression of RCN3 was also derived using the UALCAN database and found that notheness in solid or nonsolid tumor cells, RCN3 showed a high expression in immune cells and fibroblast cells.

## Abbreviations

RCC, renal cell carcinoma; UCEC, uterine corpus endometrial carcinoma; LUSC, lung squamous cell carcinoma; LUAD, lung adenocarcinoma; LIHC, liver hepatocellular carcinoma; KIRC, kidney renal clear cell carcinoma; HNSC, head and neck squamous cell carcinoma; ESCA, esophageal carcinoma; COAD, colon adenocarcinoma; CHOL, cholangiocarcinoma; BRAC, breast invasive carcinoma; GBM, glioblastoma multiforme; KICH, kidney chromophobe; CESC, cervical squamous cell carcinoma and endocervical adenocarcinoma; STAD, stomach adenocarcinoma; ACC, adrenocortical carcinoma; BLCA, bladder urothelial carcinoma; READ, rectum adenocarcinoma; SKCM, skin cutaneous melanoma; THCA, thyroid carcinoma; SARC, sarcoma; UCEC, uterine corpus endometrial carcinoma; UCS, uterine carcinosarcoma; UVM, uveal melanoma; GBM, glioblastoma multiforme; LGG, brain lower grade glioma; MESO, mesothelioma; OV, ovarian serous cystadenocarcinoma; PAAD, pancreatic adenocarcinoma; PRAD, prostate adenocarcinoma.
