# Supplementary figures and images for: Pan-Cancer Analysis of the Oncogenic and Immunological Role of RCN3: A Potential Biomarker for Prognosis and Immunotherapy
Source: Front Oncol. 2022 May 16;12:811567. doi: 10.3389/fonc.2022.811567 (PMC9149440; doi:10.3389/fonc.2022.811567)

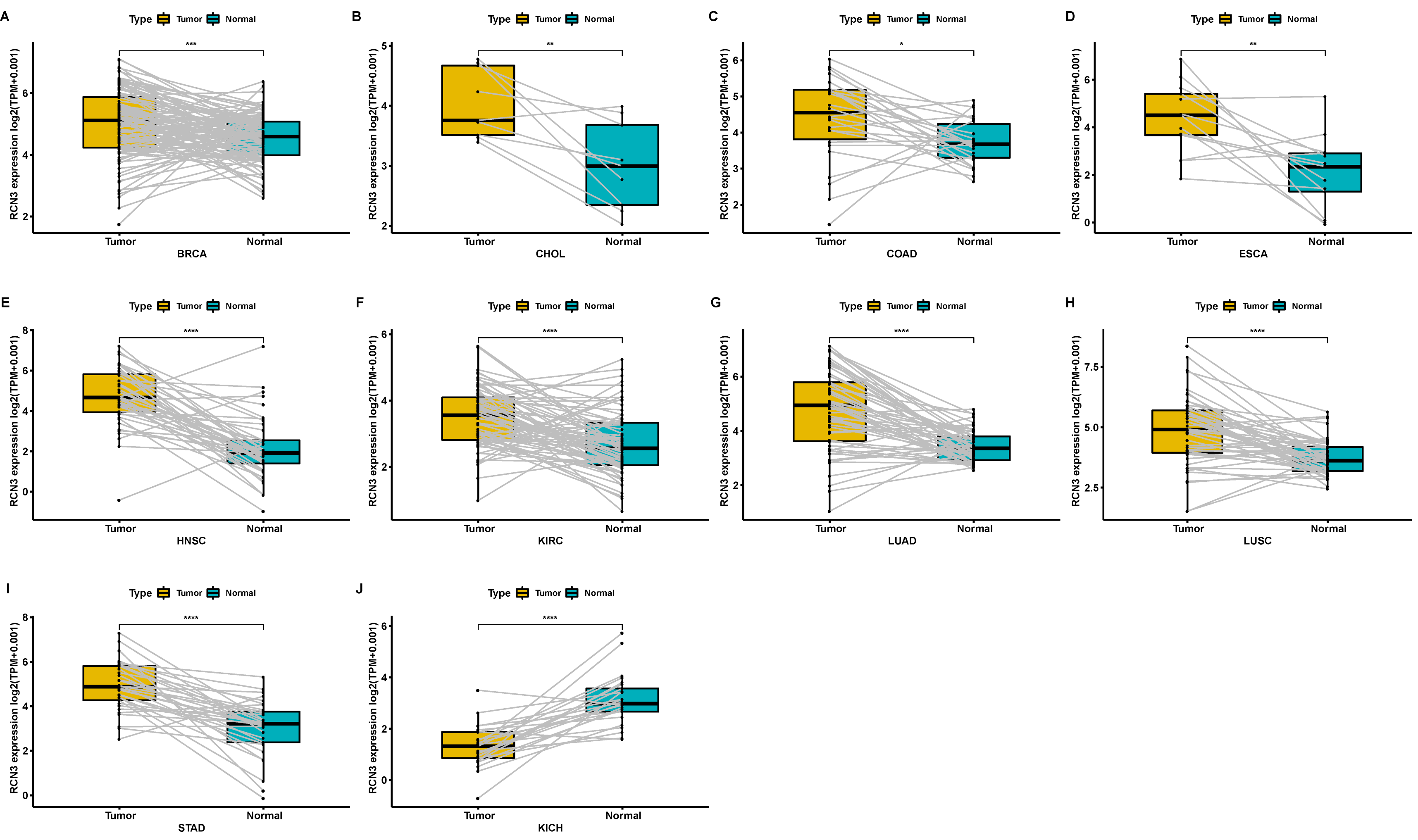

Supplement: Supplementary Figure 1 — The expression level of RCN3 in cancers. (A–J) Based on the TCGA data, the expression levels of RCN3 in different tumor tissues and matched normal tissues were analyzed using the ggpubr package. [file Image_1.tif]

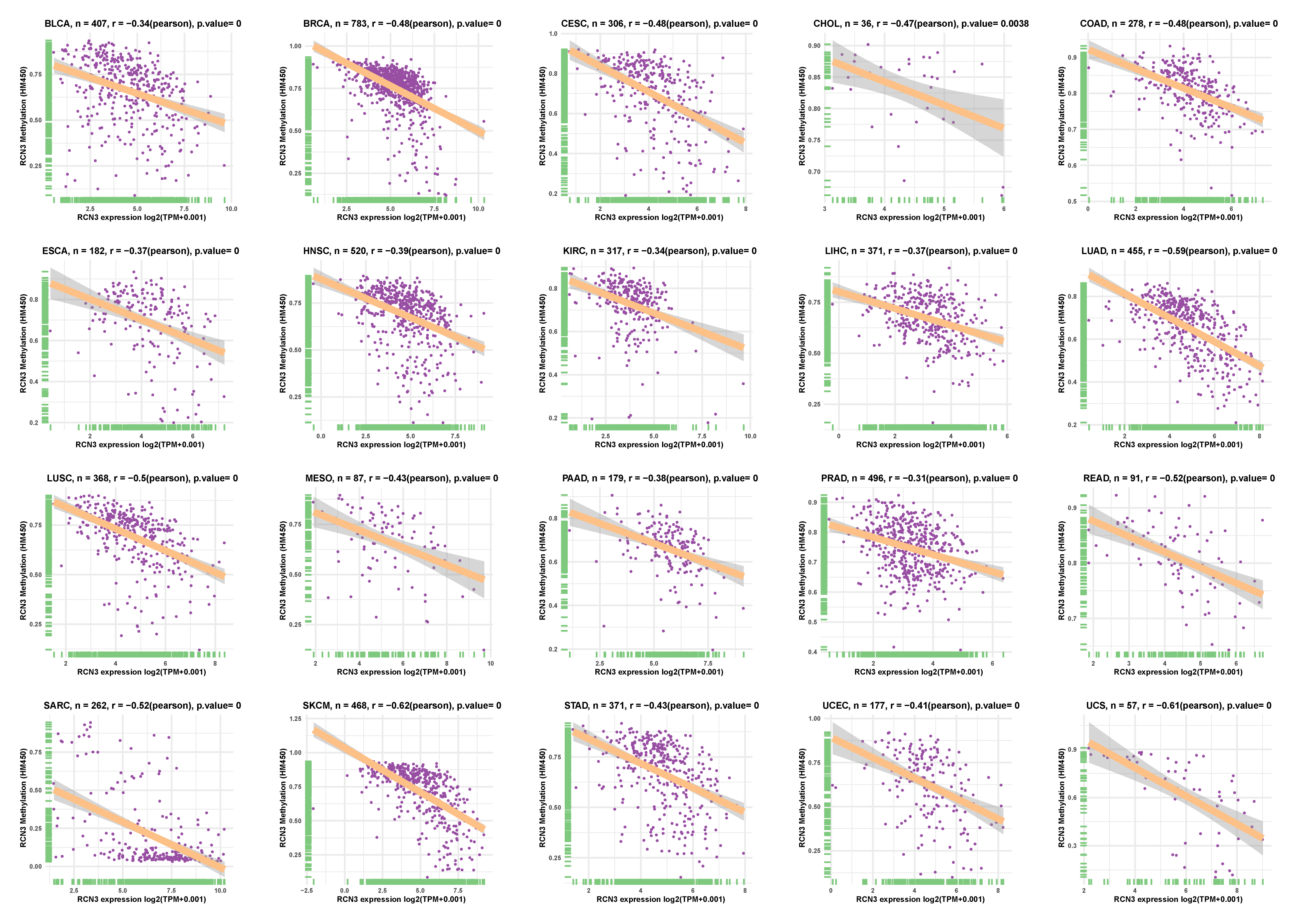

Supplement: Supplementary Figure 2 — DNA methylation of RCN3 in human cancers. Using the cBioportal tool, we explored the correlations between RCN3 expression and DNA methylation. R>0.3, P<0.05 is considered statistically significant, and only statistical results are shown here. [file Image_2.tif]

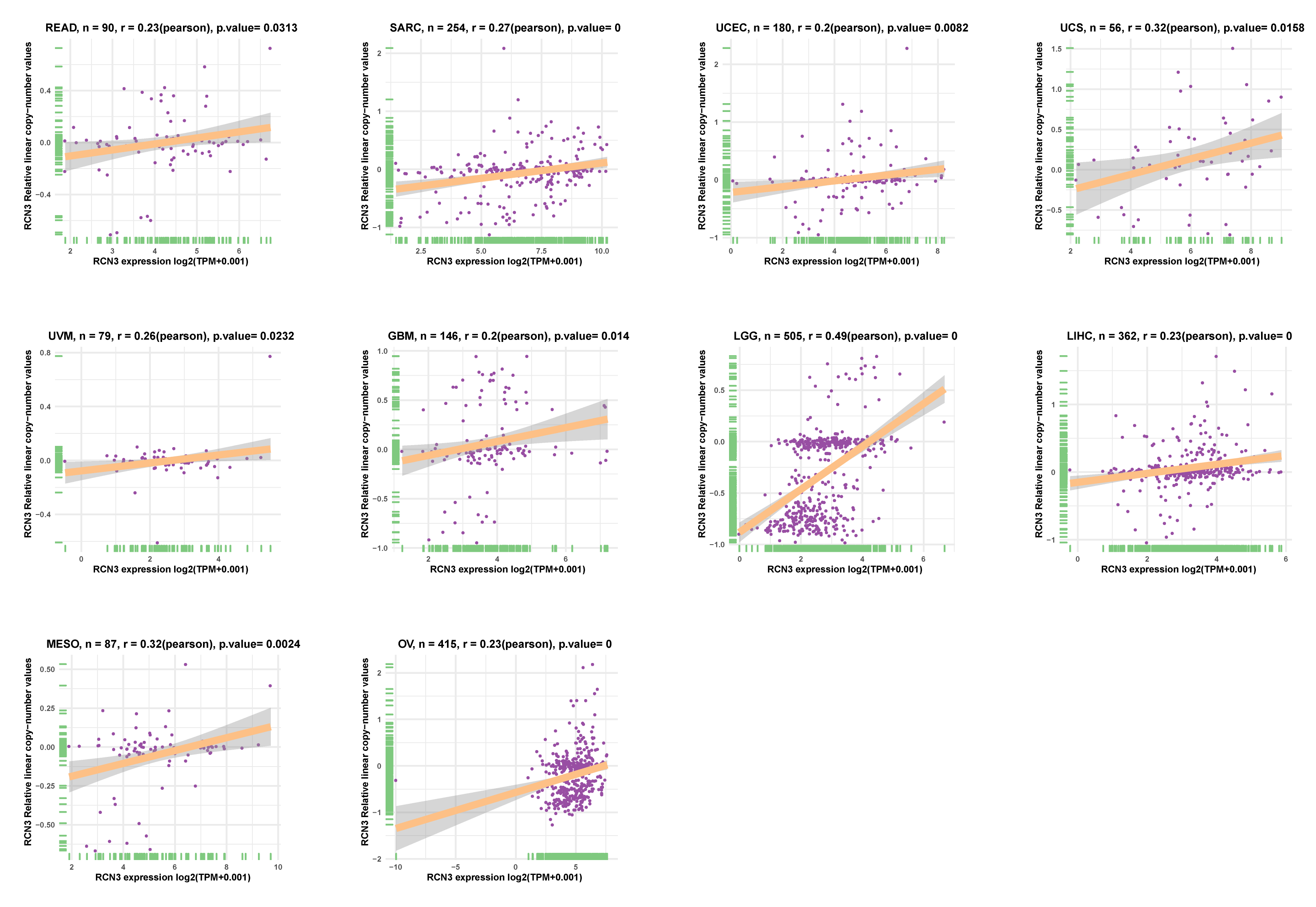

Supplement: Supplementary Figure 3 — CNA of RCN3 in human cancers. Using the cBioportal data bank, we analyzed the relationship between RCN3 expression and relative linear copy-number values. R>0.2, P<0.05 is considered statistically significant, and only statistical results are shown here. [file Image_3.tif]

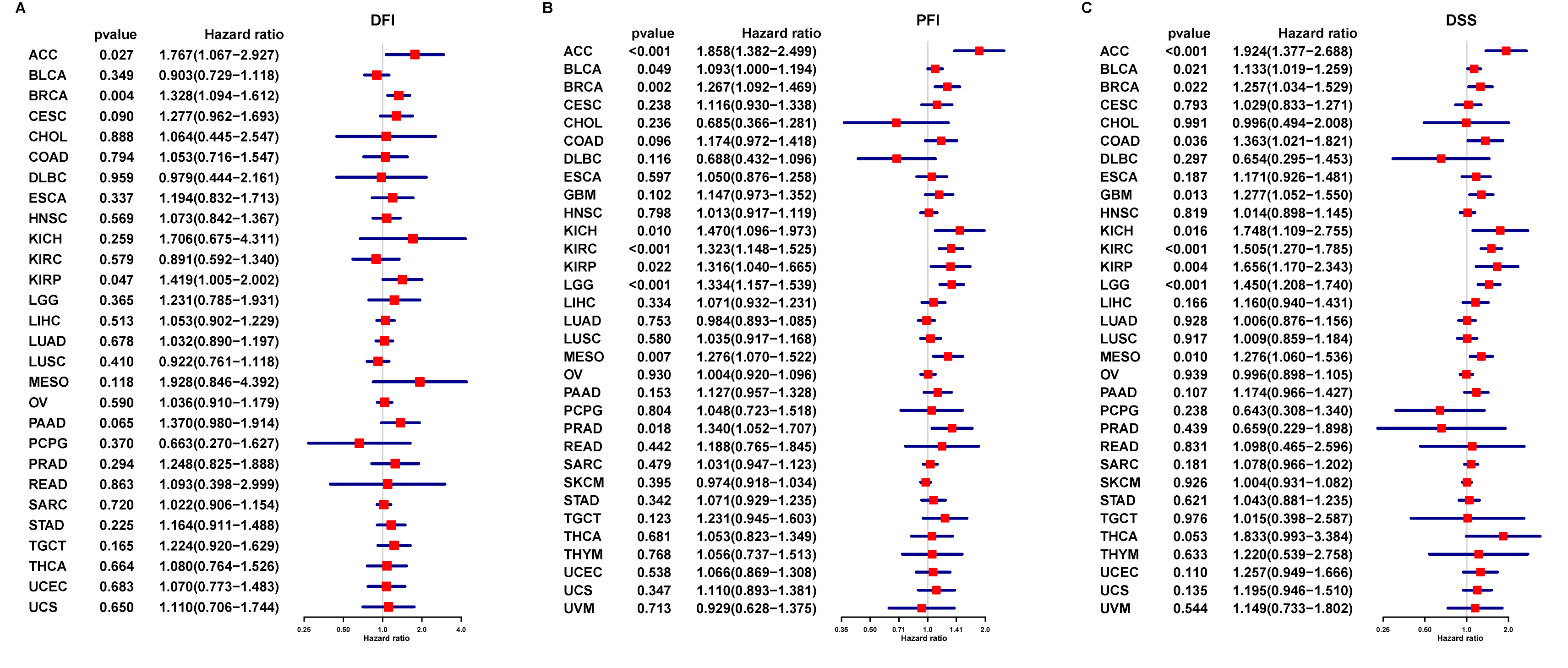

Supplement: Supplementary Figure 4 — The prognostic significance of RCN3 in pan-cancer. (A) Univariate Cox proportional-hazards regression used to quantify the relationship of RCN3 with DFI (A), PFI (B), and DSS (C). DFI, disease-free interval; PFI, progression-free interval; DSS, disease-specific survival. [file Image_4.tif]

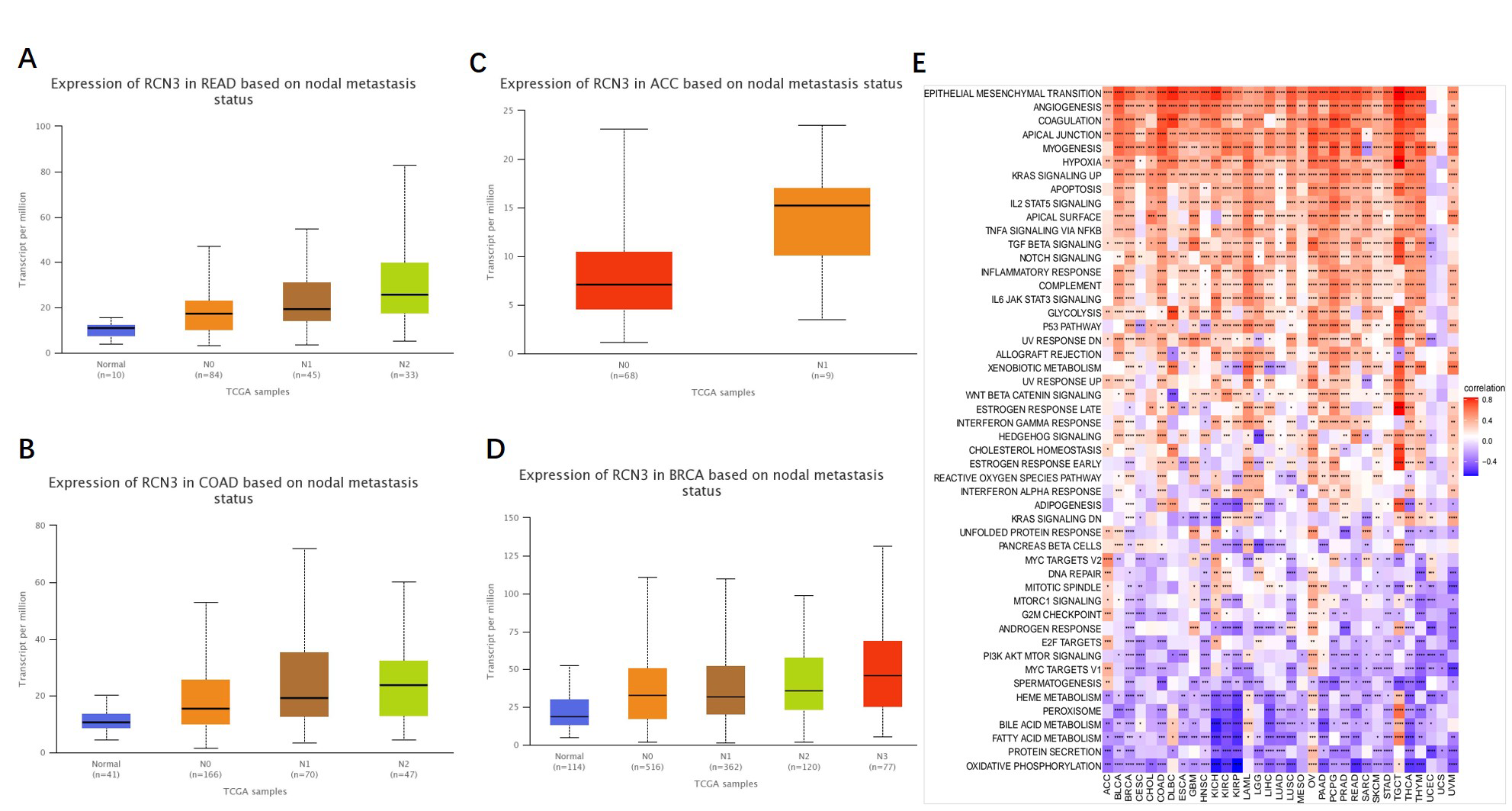

Supplement: Supplementary Figure 5 — Correlation ship of RCN3 and tumor metastasis. High expression of RCN3 share a higher nodal metastasis stage in ACC, COAD, BRCA, and READ (A–D), and a GSVA analysis of 32 cancer types showed that expression of RCN3 significantly correlated with the pathway of epithelial mesenchymal transition (EMT) (E). [file Image_5.tif]

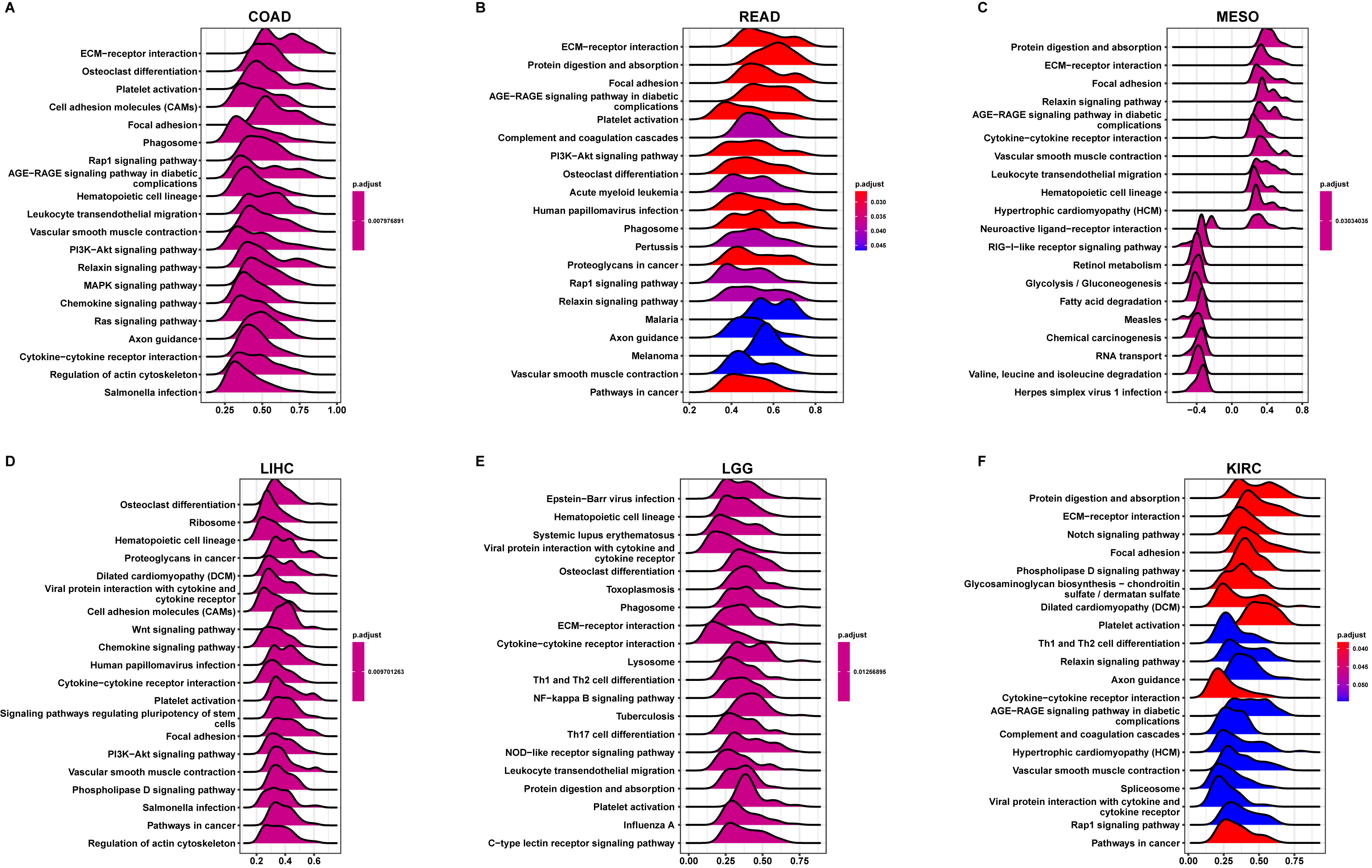

Supplement: Supplementary Figure 6 — Gene Set Enrichment Analysis (GSEA) of RCN3 in pan-cancer. (A–F) The plot shows the top 20 pathways enriched in KEGG analysis via GSEA. Significant KEGG pathways influenced by RCN3 expression in COAD, READ, MESO, LIHC, LGG, and KIRC were analyzed by GSEA. [file Image_6.tif]

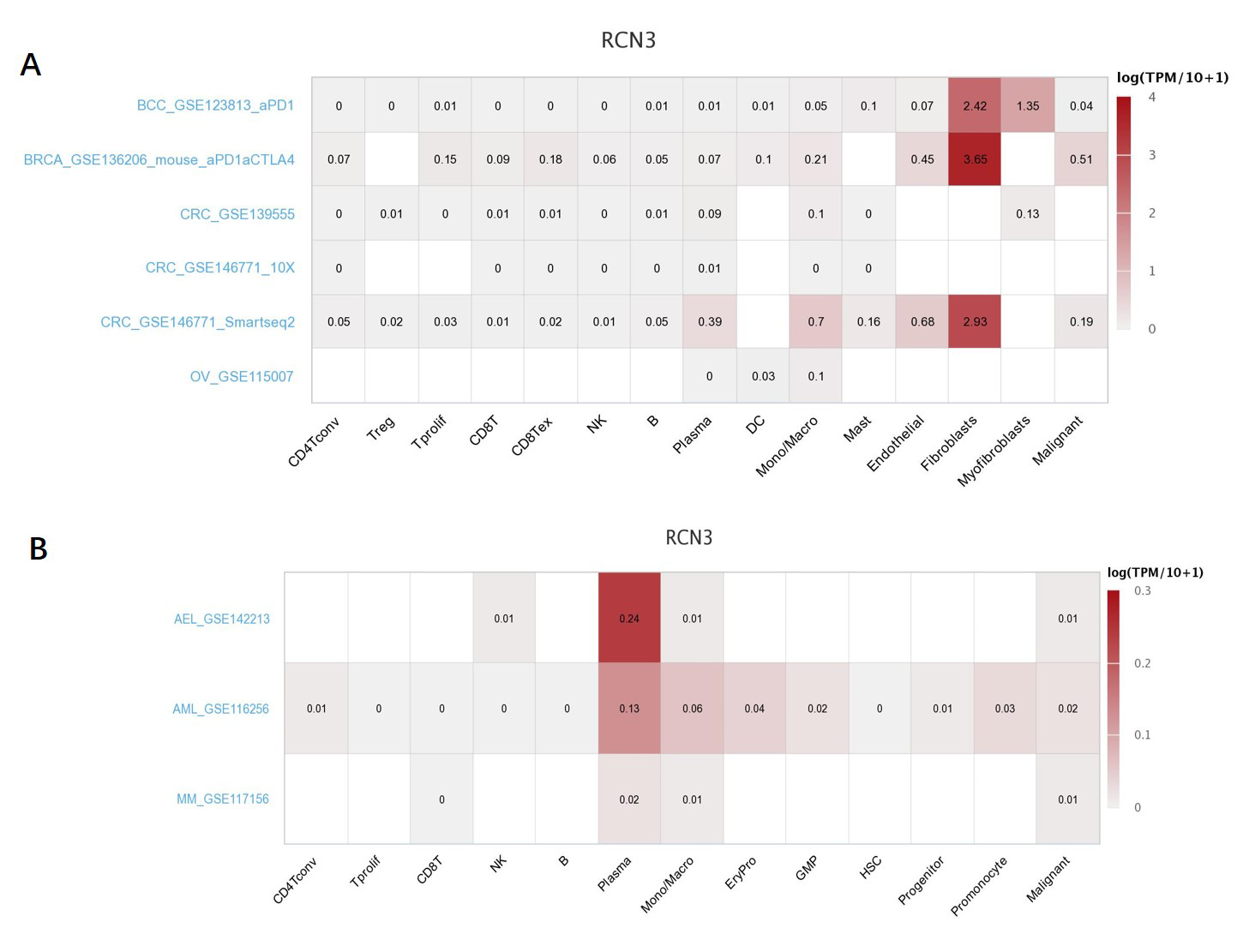

Supplement: Supplementary Figure 7 — Cell-specific expression of RCN3 was also derived using the UALCAN database and found that notheness in solid or nonsolid tumor cells, RCN3 showed a high expression in immune cells and fibroblast cells. [file Image_7.tif]
