# Supplementary material for: Pan-Cancer Analysis of the Oncogenic and Immunological Role of RCN3: A Potential Biomarker for Prognosis and Immunotherapy
Source: Front Oncol. 2022 May 16;12:811567. doi: 10.3389/fonc.2022.811567 (PMC9149440; doi:10.3389/fonc.2022.811567)
Supplement: Supplementary file 9 [file Table_2.docx]

**Table S2** The sample size of tumor tissues and normal tissues from TCGA.

| Tumor type | Full names | Sample size of tumor tissues from TCGA | Sample size of normal tissues from TCGA |
| --- | --- | --- | --- |
| ACC | Adrenocortical carcinoma | 77 | 0 |
| BLCA | Bladder Urothelial Carcinoma | 407 | 19 |
| BRCA | Breast invasive carcinoma | 1098 | 113 |
| CESC | Cervical squamous cell carcinoma and endocervical adenocarcinoma | 306 | 3 |
| CHOL | Cholangiocarcinoma | 36 | 9 |
| COAD | Colon adenocarcinoma | 288 | 41 |
| DLBC | Lymphoid Neoplasm Diffuse Large B-cell Lymphoma | 47 | 0 |
| ESCA | Esophageal carcinoma | 182 | 13 |
| GBM | Glioblastoma multiforme | 165 | 0 |
| HNSC | Head and Neck squamous cell carcinoma | 520 | 44 |
| KICH | Kidney Chromophobe | 66 | 25 |
| KIRC | Kidney renal clear cell carcinoma | 531 | 72 |
| KIRP | Kidney renal papillary cell carcinoma | 289 | 32 |
| LAML | Acute Myeloid Leukemia | 173 | 0 |
| LGG | Brain Lower Grade Glioma | 522 | 0 |
| LIHC | Liver hepatocellular carcinoma | 371 | 50 |
| LUAD | Lung adenocarcinoma | 515 | 59 |
| LUSC | Lung squamous cell carcinoma | 498 | 50 |
| MESO | Mesothelioma | 87 | 0 |
| OV | Ovarian serous cystadenocarcinoma | 427 | 0 |
| PAAD | Pancreatic adenocarcinoma | 179 | 4 |
| PCPG | Pheochromocytoma and Paraganglioma | 182 | 3 |
| PRAD | Prostate adenocarcinoma | 496 | 52 |
| READ | Rectum adenocarcinoma | 92 | 10 |
| SARC | Sarcoma | 262 | 2 |
| SKCM | Skin Cutaneous Melanoma | 469 | 1 |
| STAD | Stomach adenocarcinoma | 414 | 36 |
| TGCT | Testicular Germ Cell Tumors | 137 | 0 |
| THCA | Thyroid carcinoma | 512 | 59 |
| THYM | Thymoma | 119 | 2 |
| UCEC | Uterine Corpus Endometrial Carcinoma | 181 | 13 |
| UCS | Uterine Carcinosarcoma | 57 | 0 |
| UVM | Uveal Melanoma | 79 | 0 |
